# Supplementary material for: The Physiological Cost of Being Hot: High Thermal Stress and Disturbance Decrease Energy Reserves in Dragonflies in the Wild
Source: Biology (Basel). 2025 Jul 29;14(8):956. doi: 10.3390/biology14080956 (PMC12383770; doi:10.3390/biology14080956)

Table S1. Site name, habitat type, brief description, geographic coordinates, mean temperature (°C ± SD), maximum recorded temperature (°C), and the number of non-consecutive days during which ambient temperature was recorded using dataloggers at each study site.

Site	Habitat type	Type of water body	Latitude (°)	Longitude (°)	Mean temperature (°C)	Maximum temperature (°C)	Recording days
Arroyo Colorado	Preserved	Seasonal stream	19.507	-105.032	31.06 ± 2.01	33.55	4
Arroyo Zarco	Preserved	Seasonal stream	19.497	-105.039	31.69± 4.34	44.23	8
Charca Eje Central	Preserved	Seasonal pond	19.503	-105.038	36.50± 3.96	42.17	4
Charca EB	Preserved	Seasonal pond	19.487	-105.04	34.47± 2.41	38.31	4
Charca Antiguo Norte	Preserved	Seasonal pond	19.501	-105.048	33.93± 4.95	36.53	5
Francisco Villa	Disturbed	Permanent stream	19.385	-104.97	35.90 ± 5.08	49.92	8
Jose María Morelos	Disturbed	Seasonal stream	19.35	-104.89	33.80± 2.37	36.5	3
La Meza	Disturbed	Seasonal pond	19.6	-104.87	35.92± 3.97	46.33	6
Palmeras	Disturbed	Permanent stream	19.35	-104.89	34.01± 1.13	35.6	2
Perula	Disturbed	Seasonal pond	19.596	-105.106	38.28± 2.81	42.77	6
Río Chamela	Disturbed	Seasonal stream	19.526	-105.066	31.58± 2.66	36.7	8
El Tabaco	Disturbed	Seasonal pond	19.67	-105.151	34.23±0.51	35.2	2
Ranchitos	Disturbed	Permanent pond	19.581	-105.029	37.91± 6.75	49.63	4
Xametla	Disturbed	Seasonal pond	19.537	-105.077	37.28± 6.24	47.83	3

Table S2. Species recorded across the sampling sites. Species included in energy reserve calculation are marked with an asterisk (). *.

Preserved	
**Species**	**N**
Dythemis nigrescens	4
Erythrodiplax funerea	6
Gynacantha nervosa	2
*Macrothemis pseudimitans**	70
Micrathyria aequalis	1
*Orthemis discolor**	28
*Orthemis ferruginea**	19
Orthemis levis	2
Pantala flavescens	2
*Perithemis domitia**	9

Species	N
*Perithemis intensa**	2
*Pseudoleon superbus**	34
Disturbed	
**Species**	**N**
Dythemis nigrescens	3
Erythemis plebeja	2
*Erythrodiplax funerea**	38
Gynacantha nervosa	1
Macrothemis inacuta	5
*Macrothemis pseudimitans**	34
Macrothemis ultima	1
Micrathyria aequalis	5
*Orthemis discolor**	1
*Orthemis ferruginea**	68
Pantala flavescens	4
*Perithemis domitia**	1
*Perithemis intensa**	30
*Pseudoleon superbus**	37
Tramea onusta	3

Table S3. Selection of top competing models explaining thermal stress using maximum temperature, site condition (preserved/disturbed) and body size. The table summarizes the best-supported models based on the corrected Akaike Information Criterion (AICc). For each model, we report the number of parameters (d.f.), AICc, ΔAICc relative to the best model, and Akaike weights (w). The explanatory variables include maximum temperature (MT), site condition (SC; disturbed/preserved), body size (BS), and their interaction where applicable. Coefficients for all variables and the intercept are provided. All models included random effects accounting for sampling year and species. Models in bold indicate those with substantial support (ΔAICc < 2). The final row represents the model-averaged intercept and coefficients, along with 95% confidence intervals (CI) calculated from models with ΔAICc < 2, representing variables with substantial support.

Competing models	Explanatory variable coefficients									
	Intercept	MT	SC	BS	MT× SC	Random effects	d.f	AICc	ΔAICc	w
**MT × SC+ BS**	16.04	-0.32	-10.24	0.08	0.21	Sampling year<br>Species	8	2123	**0**	0.56
**MT × ST**	19.39	-0.32	-10.69	-	0.21	Sampling year<br>Species	7	2125	**1.67**	0.24
MT + BS	9.58	-0.17	-1.27	0.08	-	Sampling year<br>Species	7	310	2.09	0.2
	Mean intercept of the best models	95% CI								

with **ΔAICc < 2**					
17.05	[-0.49 to -0.15]	[-0.18 to -0.15]	[-0.03 to 0.15]	[0.01 to 0.41]	

Table S4. Selection of top competing models explaining energetic reserves using mean thermal stress, mean temperature and site condition (preserved/disturbed). The table summarizes the best-supported models based on the corrected Akaike Information Criterion (AICc). For each model, we report the number of parameters (d.f.), AICc, ΔAICc relative to the best model, and Akaike weights (w). The explanatory variables include mean temperature (MT), mean thermal stress (TS), site condition (SC; disturbed/preserved), and their interaction where applicable. Model coefficients are provided for all fixed variables and the intercept. All models included species as a random effect; some models also included site as a second random effect when supported. Models in bold indicate those with substantial support (ΔAICc < 2). The final row represents the model-averaged intercept and coefficients, along with 95% confidence intervals (CI) calculated from models with ΔAICc < 2, representing variables with substantial support.

Competing models	Explanatory variable coefficients									
	Intercept	TS	MT	SC	TS× SC	Random effects	d.f	AICc	ΔAICc	w
**Proteins**										
**TS+MT+SC**	2.38	-0.46	-0.28	0.04	-	Species	8	306.9	**0**	0.46
**TS× SC+MT**	2.63	-0.47	0.03	-0.33	0.03	Species	16	308.3	**1.48**	0.22
TS× SC	3.96	-0.47	-	-0.24	0.06	Species	12	309.7	2.81	0.11
	Mean intercept of the best models with **ΔAICc < 2**	95% CI								
	17.05	[-0.65 to -0.27]	[0.004 to 0.08]	[-0.51 to -0.86]	[-0.04 to 0.06]					
**Lipids**										
**TS× SC+MT**	-1.04	-0.56	0.04	-0.5	0.09	Species	7	430.4	**0**	0.39
**TS×SC**	0.41	-0.57	-	-0.41	0.12	Species	6	430.8	**0.35**	0.33
**TS+ SC+MT**	-1.82	-0.53	0.062	-0.35	-	Species	6	431.8	**1.4**	0.19

	Mean intercept of the best models with **ΔAICc < 2**	95% CI								
	-0.68	[-0.74 to -0.37]	[-0.03 to 0.09]	[-0.74 to - 0.37]	[-0.03 to 0.21]					
**Thoracic mass**										
**TS×SC+MT**	3.09	0.45	-	0.26	-0.09	Species,Site	7	199.9	**0**	0.45
**TS×SC**	3.97	0.45	-0.02	0.33	-0.08	Species,Site	6	201.3	**1.38**	0.22
TS	3.23	0.41	-	-	-	Species,Site	5	202.3	2.38	0.13
	Mean intercept of the best models with **ΔAICc < 2**	95% CI								
	4.79	[0.29 to 0.60]	[-0.04 to 0.03]	[-0.009 to 0.59]	[-0.16 to -0-01]					

Figure S1. Predicted protein content in relation to mean temperature, based on model averaging across the two best-supported models (ΔAICc < 2). Shaded areas represent 95% confidence intervals. All values were back-tranformed from the logarithmic scale.

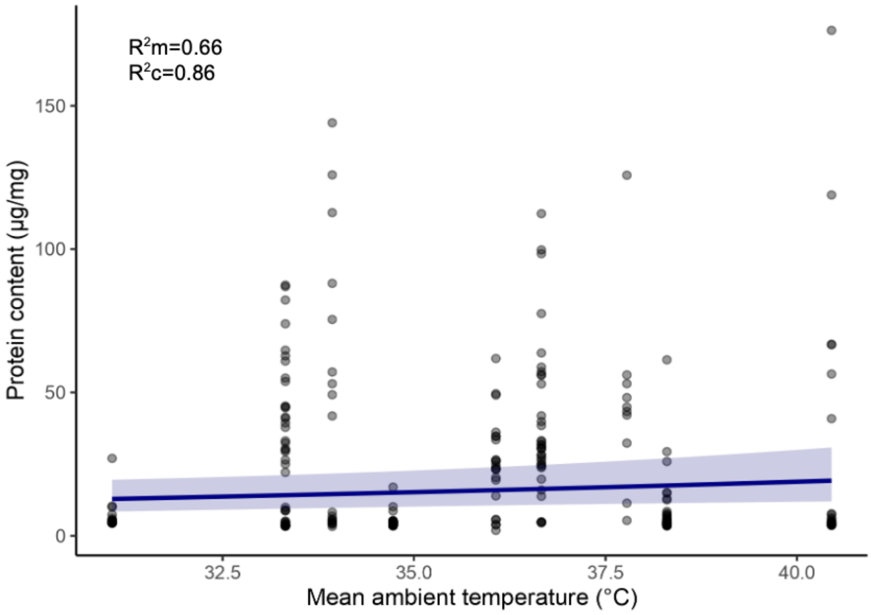

Supplement: Supplementary file 1 [file biology-14-00956-s001.zip › biology-3669880-supplementary.pdf]
